# Performance of intensive care unit severity scoring systems across different ethnicities in the USA: a retrospective observational study

**DOI:** 10.1016/S2589-7500(21)00022-4

**Published:** 2021-04

**Authors:** Rahuldeb Sarkar, Christopher Martin, Heather Mattie, Judy Wawira Gichoya, David J Stone, Leo Anthony Celi

**Affiliations:** Department of Respiratory Medicine (R Sarkar MPH) and Department of Critical Care (R Sarkar), Medway NHS Foundation Trust, Gillingham, Kent, UK; Faculty of Life Sciences, King’s College London, London, UK (R Sarkar); UCL Institute for Health Informatics, London, UK (C Martin PhD); Crystallise, Essex, UK (C Martin); Department of Biostatistics, Harvard T H Chan School of Public Health, Boston, MA, USA (H Mattie PhD, L A Celi PhD); Interventional Radiology and Informatics, Department of Radiology and Imaging Sciences, Emory University, Atlanta, GA, USA (J W Gichoya MD); Department of Anesthesiology (D J Stone MD), Department of Neurosurgery (D J Stone), and Center for Advanced Medical Analytics (D J Stone), University of Virginia School of Medicine, Charlottesville, VA, USA; Laboratory for Computational Physiology, Massachusetts Institute of Technology, Cambridge, MA, USA (L A Celi); Division of Pulmonary, Critical Care and Sleep Medicine, Beth Israel Deaconess Medical Center, Boston, MA, USA (L A Celi)

## Abstract

**Background:**

Despite wide use of severity scoring systems for case-mix determination and benchmarking in the intensive care unit (ICU), the possibility of scoring bias across ethnicities has not been examined. Guidelines on the use of illness severity scores to inform triage decisions for allocation of scarce resources, such as mechanical ventilation, during the current COVID-19 pandemic warrant examination for possible bias in these models. We investigated the performance of the severity scoring systems Acute Physiology and Chronic Health Evaluation IVa (APACHE IVa), Oxford Acute Severity of Illness Score (OASIS), and Sequential Organ Failure Assessment (SOFA) across four ethnicities in two large ICU databases to identify possible ethnicity-based bias.

**Methods:**

Data from the electronic ICU Collaborative Research Database (eICU-CRD) and the Medical Information Mart for Intensive Care III (MIMIC-III) database, built from patient episodes in the USA from 2014–15 and 2001–12, respectively, were analysed for score performance in Asian, Black, Hispanic, and White people after appropriate exclusions. Hospital mortality was the outcome of interest. Discrimination and calibration were determined for all three scoring systems in all four groups, using area under receiver operating characteristic (AUROC) curve for different ethnicities to assess discrimination, and standardised mortality ratio (SMR) or proxy measures to assess calibration.

**Findings:**

We analysed 166 751 participants (122 919 eICU-CRD and 43 832 MIMIC-III). Although measurements of discrimination were significantly different among the groups (AUROC ranging from 0·86 to 0·89 [p=0·016] with APACHE IVa and from 0·75 to 0·77 [p=0·85] with OASIS), they did not display any discernible systematic patterns of bias. However, measurements of calibration indicated persistent, and in some cases statistically significant, patterns of difference between Hispanic people (SMR 0·73 with APACHE IVa and 0·64 with OASIS) and Black people (0·67 and 0·68) versus Asian people (0·77 and 0·95) and White people (0·76 and 0·81). Although calibrations were imperfect for all groups, the scores consistently showed a pattern of overpredicting mortality for Black people and Hispanic people. Similar results were seen using SOFA scores across the two databases.

**Interpretation:**

The systematic differences in calibration across ethnicities suggest that illness severity scores reflect statistical bias in their predictions of mortality.

## Introduction

Severity scoring systems are used in the intensive care unit (ICU) to perform severity adjustment for the purposes of benchmarking and research.^1^ These systems have generally been assumed to be fair and objective in terms of their use across different ethnicities. However, although such models can perform differently among disparate geographical populations or between different centres,^2^ the assumption of scoring neutrality among ethnicities within a given population has not been closely examined.

Disparities in ICU outcomes might result from pre-admission clinical factors, socioeconomic determinants, the quality of ICU care, and cultural practices.^3,4^ Another possible source of disparity emanates from the use of biased algorithms.^5–8^ The current COVID-19 pandemic raises two intersecting issues that demand closer evaluation. First, higher mortalities have been observed in particular ethnic populations, specifically African American people, when compared with White populations.^9^ Second, severity scores have been proposed by professional societies and various policy groups to be incorporated into triage systems for potential scarce resource allocation.^10,11^ It is therefore imperative to determine whether biased scoring systems could be adding to existent baseline disparities in health care.

Many different risk scoring models have been used in clinical medicine, including critical care. The latest model of the Acute Physiology and Chronic Health Evaluation (APACHE) scoring system, APACHE IVa, was developed using data from 104 ICUs in 45 US-based hospitals using 142 patient variables. The model uses the worst values in the first APACHE day (ie, within the first 24 h of admission) of the patient’s ICU stay to generate a risk score for hospital and ICU mortality and length of stay.^12^ The Oxford Acute Severity of Illness Score (OASIS) was developed from 81 087 admissions from 86 ICUs in the USA, using ten variables collected in the first 24 h of ICU stay.^13^ The Sequential Organ Failure Assessment (SOFA) score was developed based on expert opinion, incorporating organ function scores from six organ systems to characterise severity state in sepsis, but has been repurposed to predict patient outcomes.^14^ In addition to acute physiological measurements, APACHE IVa adjusts for age, chronic health condition, admission information, and admission diagnosis. OASIS adjusts for age, pre-ICU length of stay, and whether the admission was an emergency or elective. SOFA does not adjust for factors outside of the six organ function scores and was not specifically developed for mortality prediction, unlike APACHE IVa and OASIS.

In this retrospective observational study, we examined the performance of these three severity scoring prediction models—APACHE IVa, OASIS, and SOFA—in two large, publicly available ICU databases (electronic ICU Collaborative Research Database [eICU-CRD] and Medical Information Mart for Intensive Care III [MIMIC-III]).

## Methods

### Databases

The eICU-CRD was derived from the eICU telehealth system.^15^ This system was designed to complement on-site ICU teams with remote support. The data include more than 200 000 discharged patient episodes across 335 ICUs at 208 hospitals (both academic and non-academic) in the USA during 2014–15. Patient demographics available in the eICU-CRD database include age, sex, ethnicity, vital signs, diagnoses, laboratory measurements, clinical history, problem lists, APACHE IVa scores, and treatments.

MIMIC-III is a publicly available database consisting of more than 60 000 ICU admissions to the Beth Israel Deaconess Medical Centre (BIDMC; Boston, MA, USA) between 2001 and 2012.^16^ MIMIC-III incorporates OASIS as a mortality prediction model.

Admission SOFA scores were computed in both databases. Mortality in all groups was calculated at multiple SOFA cutoffs, with SOFA score categories of 0–7, 8–11, and more than 11. The categories were based on what has been proposed for COVID-19 ventilator allocation guidelines to examine the model performance in the proposed triage categories.^10^

The US Federal guidance classifies race into five categories (American Indian or Alaska Native, Asian, Black or African American, Native Hawaiian or other Pacific Islander, and White), and ethnicities into two categories (Hispanic or Latino and not Hispanic or Latino).^17^ For this Article, we defined ethnicity on the basis of entries made in the demographic sections of the respective databases. The ethnicities included in the analyses were Black, Asian, Hispanic, and White. Native American people were excluded due to the much smaller sample size compared with the other ethnicities (n=946 [0·70%] in the eICU-CRD and n=57 [0·11%] in the MIMIC-III database). Patient episodes with a non-specific or unknown ethnicity category were excluded. Patients with missing survival data, erroneous or missing prediction scores, missing ethnicity data, and those younger than 16 years or older than 90 years were excluded from the analyses.

Ethnicity information was available in both the databases. This information is typically entered by an administrator, who asks the patient or family member which ethnicity they identify with, or is obtained from previously available records.

Research using the eICU-CRD is exempt from institutional review board approval due to the retrospective design, lack of direct patient intervention, and the security schema, for which the re-identification risk was certified as meeting safe harbour standards by an independent privacy expert (Privacert, Cambridge, MA, USA; Health Insurance Portability and Accountability Act Certification number 1031219–2). The data in the MIMIC-III database has been previously de-identified, and the institutional review boards of the Massachusetts Institute of Technology (number 0403000206) and BIDMC (number 2001-P-001699/14) both approved the use of the database for research. No informed consent was obtained, and all available data in the databases were anonymous.

### Statistical analysis

Discrimination was determined by the area under receiver operating characteristic (AUROC) curve for different ethnicities. Mortality during hospital stay encompassing the ICU admissions analysed was the outcome of interest. SOFA score was analysed in both databases, APACHE IVa was used as a predictor in the eICU-CRD, and OASIS was used as a predictor in the MIMIC-III database. Statistical significance of differences of key variables across ethnic groups was tested using regression of dummy indicator variables.

Calibration was evaluated using standardised mortality ratio (SMR) for APACHE IVa and OASIS. Because predicted mortality for a given SOFA score for an individual patient cannot be calculated, SMR could not be specifically calculated for SOFA. Instead, observed mortality for each ethnic group was compared to the mortality rate in the overall population in that SOFA score category in order to provide an evaluation of comparative outcomes among ethnic groups.

To further characterise model performance in the context of sicker patient populations, an additional calibration analysis was performed across risk grades of 0–5%, more than 5–10%, more than 10–20%, more than 20–50%, and more than 50%, based on APACHE IVa and OASIS in the eICU-CRD and MIMIC-III patients (appendix p 2).

The statistical analyses were done in R, version 4.0.0. The packages used included rsq (partial R2), version 2.0; ems (SMR), version 1.3.2; dplyr (data handling and summarising), version 1.0.0; and pRoc. Stata, version 14, was used for comparison of AUROC between groups using the Roccomp function.

### Role of the funding source

There was no funding source for this study.

## Results

**Figure 1:**
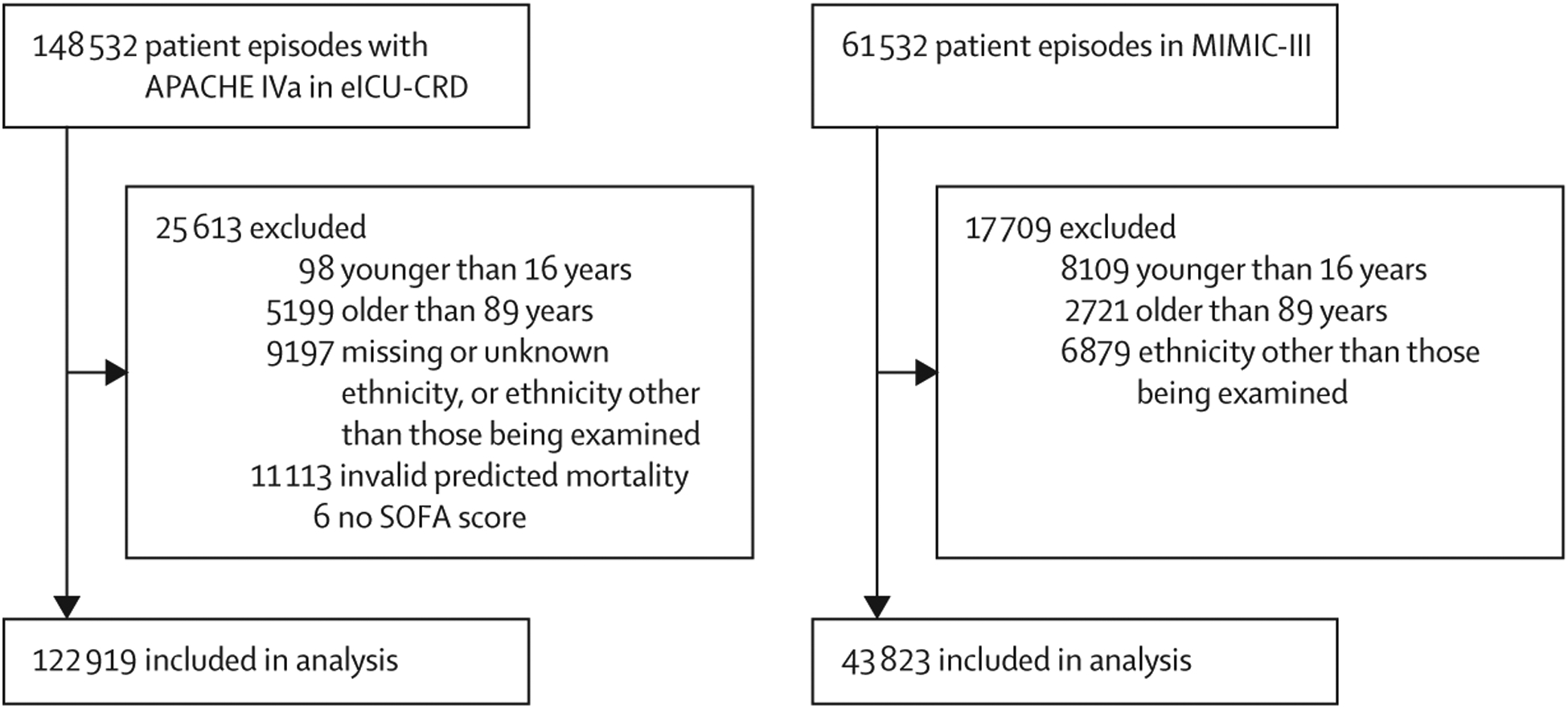
Study flow Excluded patients in both databases; the exclusions have been made in the sequence specified in the diagram. APACHE IVa=Acute Physiology and Chronic Health Evaluation IVa. eICU-CRD=electronic intensive care unit Collaborative Research Database. MIMIC-III=Medical Information Mart for Intensive Care III. SOFA=Sequential Organ Failure Assessment.

The distribution and characteristics of patients are shown in table 1. 43 322 patients with missing or unknown ethnicity, ethnicities other than the four being examined, outside the age range of 16–89 years, or without a valid model-predicted mortality (required for SMR calculation) were excluded (figure 1). The total numbers of ICU admissions included in the final analysis were 122 919 (82·8% of all episodes) in the eICU-CRD and 43 823 (71·2% of all episodes) in the MIMIC-III database.

Black people and Hispanic people were younger than patients of other ethnicities (p<0·0001). Mean prediction scores were similar across the groups. Predicted hospital mortalities across ethnicities were in the 11–12% range in the eICU-CRD and 11–14% in the MIMIC-III database, whereas observed mortalities were 8–9% in the eICU-CRD and 7–13% in the MIMIC-III database, indicating that both models overestimated hospital mortality.

**Figure 2:**
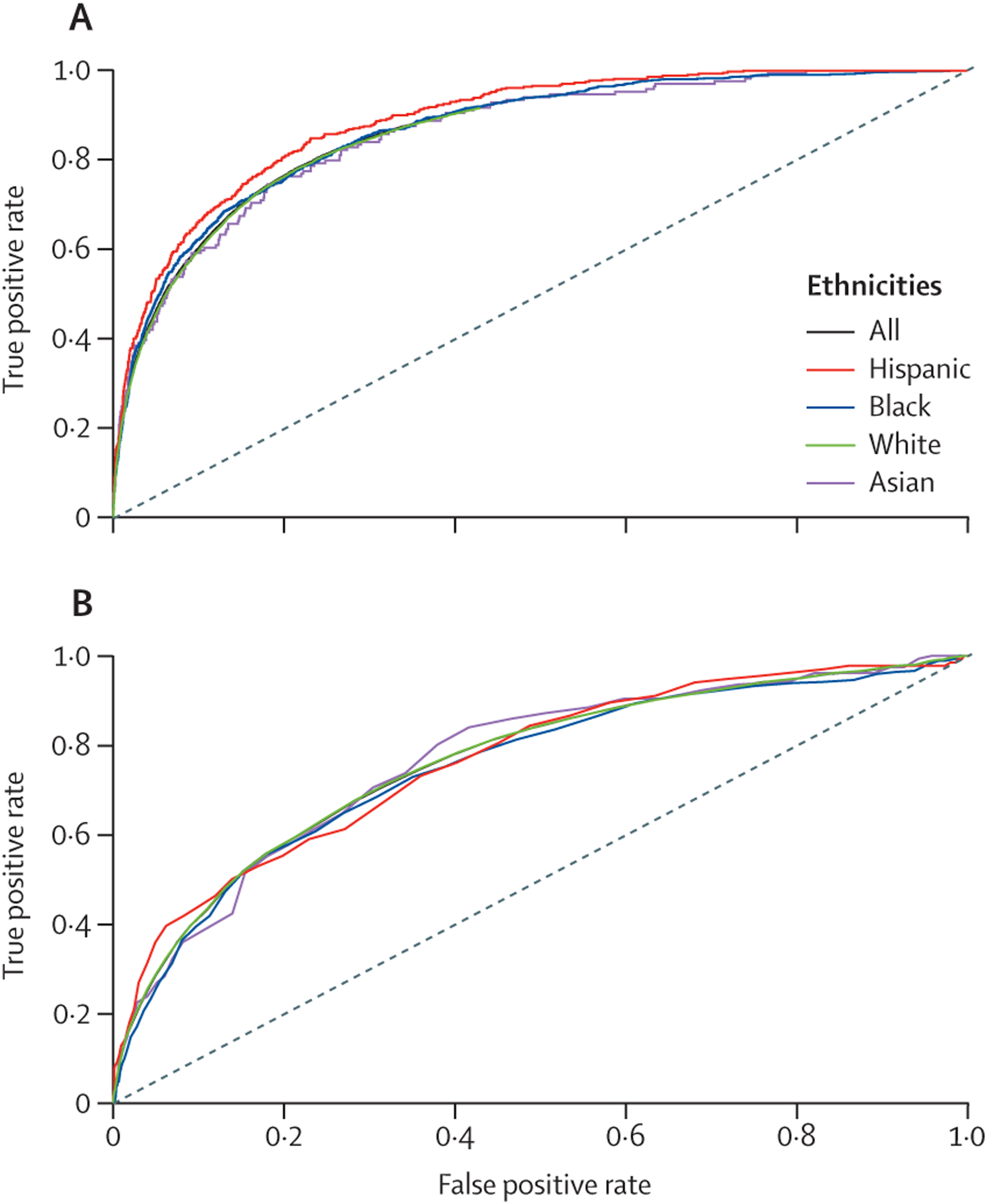
ROC for predicted hospital mortality by ethnicity in the eICU-CRD (A) ROC for the APACHE IVa-predicted hospital mortality in the eICU-CRD by ethnicity. The AUROC for all was 0·86, Hispanic 0·89, Black 0·87, White 0·86, and Asian 0·86. (B) ROC for the OASIS-predicted hospital mortality in the MIMIC-III database by ethnicity. The AUROC for all was 0·76, Hispanic 0·76, Black 0·75, White 0·76, and Asian 0·77. ROC=receiver operating curve. APACHE IVa=Acute Physiology and Chronic Health Evaluation scoring system IVa. eICU-CRD=electronic intensive care unit Collaborative Research Database. AUROC=area under receiver operating characteristic. OASIS=Oxford Acute Severity of Illness Score. MIMIC-III=Medical Information Mart for Intensive Care III.

Tests for discrimination showed that the APACHE IVa model performed well across all ethnicities in the eICU-CRD, with an AUROC of 0·89 for Hispanic patients, 0·87 for Black patients, 0·86 for Asian patients, and 0·86 for White patients (figure 2; appendix p 4). Across-group differences in the AUROC were statistically significant (p=0·016). In the MIMIC-III database, the AUROC was 0·76 in the Hispanic group, 0·75 in the Black group, 0·76 in the White group, and 0·77 in the Asian group, displaying non-significant across-group differences (p=0·85).

**Figure 3:**
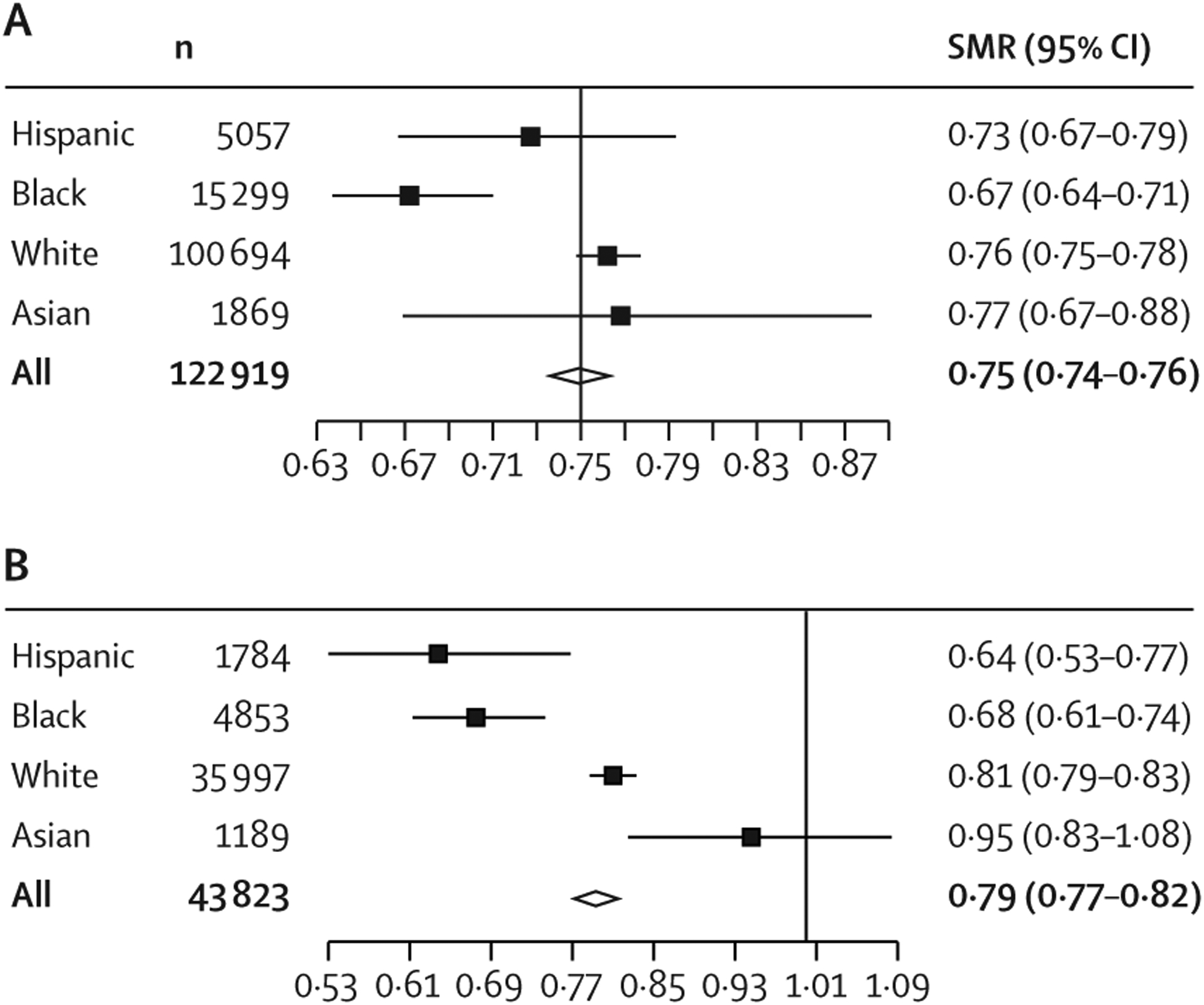
SMR for APACHE score in the eICU-CRD and OASIS score in MIMIC-III across ethnicities (A) Forest plot for SMRs from the eICU-CRD for mortality predicted by APACHE IVa. (B) Forest plot for SMRs for different ethnicities from the MIMIC-III database for predicted mortality determined by OASIS. SMR=standardised mortality ratio. eICU-CRD=electronic intensive care unit Collaborative Research Database. APACHE IVa=Acute Physiology and Chronic Health Evaluation scoring system IVa. OASIS=Oxford Acute Severity of Illness Score.

**Figure 4:**
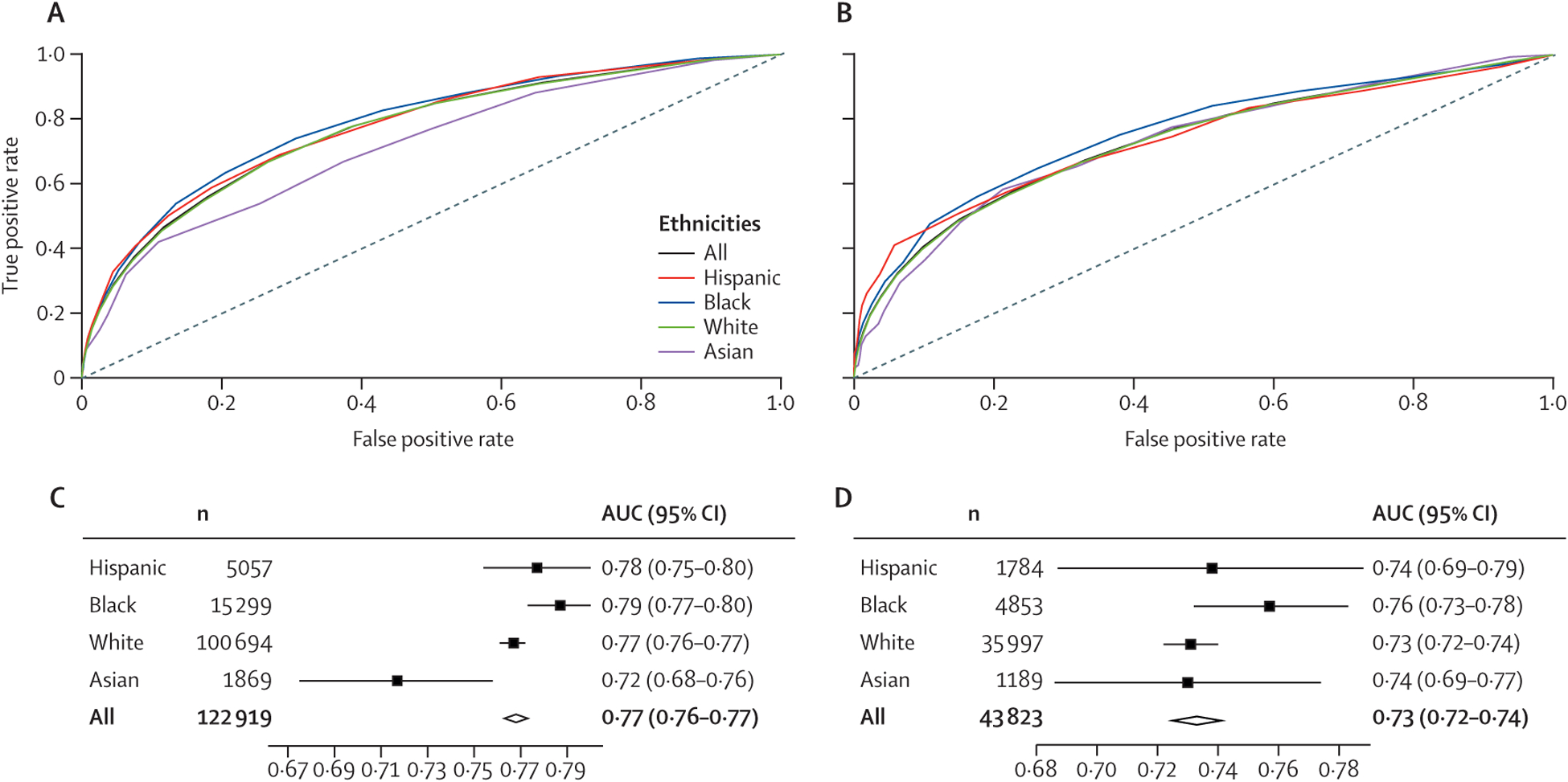
ROC curves for forest plots for predicted mortality by SOFA score in eICU-CRD and MIMIC-III (A) ROC plots for all ethnicities in the eICU-CRD for SOFA score performance in hospital mortality prediction. The AUROC for all was 0·77, Hispanic 0·78, Black 0·79, White 0·77, and Asian 0·72. (B) ROC plots for all ethnicities in MIMIC-III for SOFA score performance in hospital mortality prediction. The AUROC for all was 0·73, Hispanic 0·74, Black 0·76, White 0·73, and Asian 0·73. (C) Forest plot for AUROCs in different ethnicities in the eICU-CRD for performance of SOFA score with 95% CIs. (D) Forest plot for AUROCs in different ethnicities in MIMIC-III for performance of SOFA score with 95% CIs. AUC=area under the curve. AUROC=area under receiver operating characteristic. eICU-CRD=electronic intensive care unit Collaborative Research Database. MIMIC-III=Medical Information Mart for Intensive Care-III. SOFA=Sequential Organ Failure and Assessment.

10 562 deaths were observed in the eICU-CRD, compared with 14 097 deaths predicted by the APACHE IVa model (appendix p 5). This overprediction of mortality was also observed in the MIMIC-III database, with 4847 deaths observed compared with 6113 expected deaths predicted by the OASIS model. The APACHE IVa model was least accurate for predicting hospital mortality in Black people (SMR 0·67) and most accurate in Asian people (SMR 0·77; figure 3; appendix p 5). The SMRs for Black people and White people in the eICU-CRD using APACHE IV were statistically significantly different (p<0·0001) using two-sample test of proportions. OASIS was least accurate in Hispanic people (SMR 0·64) and Black people (SMR 0·67), and most accurate in Asian people (SMR 0·95). SMRs across the group were significantly different; however, this was not true of all pairwise comparisons. There appeared to be two distinct groupings: one comprising the Hispanic and Black groups, and another comprising the Asian and White groups, with the Hispanic and Black group displaying significantly worse calibration than the Asian and White group, although this only reached statistical significance in the MIMIC-III database on inspection of the CIs in the forest plots in figure 3. Notably, the White and Black groups were distinctly separated from one another, with lower SMRs for Black groups in both databases. When using SOFA score, discrimination was similar between the two databases (AUROC 0·77 for eICU-CRD *vs* 0·73 for OASIS) and across ethnicities in both databases, with the exception of the Asian group in the eICU-CRD for which the AUROC was considerably lower (figure 4). For the other three groups in this database, AUROCs ranged from 0·77 to 0·79, whereas in the MIMIC-III database, AUROCs for each ethnicity ranged from 0·73 to 0·76 (figure 4). As noted earlier, usual SMRs could not be calculated to determine calibrations for SOFA; however, we observed the same phenomenon of a lower observed mortality for a given risk score category in Black people (and less so for Hispanic people), compared with White people and Asian people across several of the score categories (table 2; appendix p 5). SOFA mortalities also seemed to differ in the databases for the same scoring category within a given ethnic group (table 2).

## Discussion

In this comparative study of the performance of ICU mortality prediction models in different ethnicities, we show that while there was a statistically significant difference across the AUROCs, there was no systematic pattern to the difference in the discriminative performances of APACHE IVa, SOFA, and OASIS. However, OASIS, APACHE IVa, and SOFA overpredicted mortality in all ethnic groups. This poor calibration was particularly notable in the Black and Hispanic groups. There was a statistically significant difference between the SMRs of White people and Black people for both APACHE IVa (p<0·0001) and OASIS (p<0·0001), and a statistically significant difference between White people and Hispanic people for OASIS (p<0·0001). Asian people were statistically different from Black people (p<0·0001) and Hispanic people (p<0·0001) in OASIS only (figure 3). Although not designed for mortality prediction, SOFA performed reasonably well in terms of discrimination, with the exception of the somewhat aberrant AUROC in the Asian group in the eICU-CRD. The relative mortality risks in Hispanic and Black groups were lower in the two databases for low to moderately high SOFA scores. This difference must be taken into consideration when SOFA is used for prognostication and triage decisions in the ICU.

Importantly, although it is reassuring that all scores were better calibrated in the sicker population (ie, those with SOFA scores ≥12), it is of concern that in mild to moderate risk categories, including mid-range SOFA scores, calibration was poor in the Black and Hispanic groups, who are more likely to come from socioeconomic backgrounds associated with poor social determinants of health, compared with the Asian and White groups. Although calibrations were less disparate at the highest scores of more than 11 (indicating very poor prognoses), the mortality ratio for Black people was still more than 10% lower than that of White people and Asian people in the ICU database at this level.

These findings have potential repercussions for some guidelines^10,11^ on the appropriation of limited ICU resources during the COVID-19 pandemic. For a persistent SOFA score of 8–11 after 48 or 120 h, evaluation of treatment continuation has been proposed to be necessary.^10,11^ If SOFA does overpredict mortality in that score range, then this form of decision making could be misguided. The same guidelines from New York and Michigan (USA) have used a level of 12 as a potential cutoff for admission or continued ICU care. The reason Black and Hispanic groups have shown such inaccurately high mortality predictions in this study needs to be elucidated. Such inaccurate predictions are concerning, particularly if treatment is withheld or care withdrawn on the basis of a false high predicted mortality.

Precise calibration is important if these systems are to be used for care decisions in individual patients. Triage decisions related to patient admission, management (including discontinuation of treatment), and discharge from the ICU are potentially subjective and vulnerable to bias. Scoring systems might be applied to these decisions to, in theory, introduce a greater level of objectivity and fairness when resources are critically limited. However, if the systems themselves are biased, then their use for these purposes will systemically imprint and effectively endorse existing inequities. Another important point is the use of prediction models based on a single timepoint, because this might not always capture an individual’s potential to respond to a proposed treatment. However, in real-world decision making, especially in a resource-constrained scenario, all that is available to the clinician or a triage official is a snapshot type of risk prediction tool.

Although a temporal drift in model performance might explain low SMRs in all the ethnic groups, it is not clear why these scoring systems produce ethnically consistent patterns of poor calibration. The drift should have occurred equally in all ethnicities over time, if the models performed equally at all timepoints in all ethnicities. Based on the results of studies done in 2020, it is unlikely that Black and Hispanic patients received relatively better care.^18,19^ It is also unlikely that an identical physiological phenotype represents a different disease trajectory in those groups. An implicit assumption of scoring systems is that patients have the same baseline states and that the scores represent the same degree of deviation from that baseline state. However, Black people and Hispanic people admitted to ICU with the same severity scores as White people and Asian people, might actually be exhibiting a smaller change from their baseline status. For example, a population with a higher prevalence of chronic organ failure (eg, baseline elevations in serum creatinine or bilirubin) could show SOFA scores that do not accurately portray their acute physiological status. Deliberato and colleagues^20^ have shown that patients with obesity—for which African American and Hispanic populations are at increased risk^21^—might be similarly misclassified with regard to illness severity, with absolute physiological measurements on ICU admission giving the appearance of a more abnormal baseline state compared with patients with a lower body-mass index.^20^ Chronic disease burden has also been suggested to contribute more towards mortality in patients who are critically ill.^22^ Given that the Hispanic and Black groups were younger than the White and Asian groups in both databases, it is possible that they had a low chronic disease burden, resulting in a lower contribution of chronic disease towards mortality risk for the similar acute physiological profile.

In a world without bias and health disparities, only patient and disease factors would determine case-mix and clinical outcomes in the ICU. However, studies have repeatedly shown that this is not the case.^18,19^ Our detection of inadvertent, but undeniable, bias in severity scores would seem to indicate that it is time to develop scoring systems that are more precise than the current one-size-fits-all systems. This will admittedly pose a challenge, but one that is achievable as more data accumulate for varying patient cohorts and contexts. In response to this need, there is a movement across the critical care community to make mortality risk prediction models more dynamic and useful in real time, often based on data collected from electronic health records.^23–27^ Notably, around 70% of the patients were White in the training and validation datasets for APACHE IVa and OASIS models. More diverse ethnic representation of patients during model development will help reduce potential bias. Attention must be paid to relevant sociodemographic factors while developing the models. Especially with the potential resource limitations arising in the COVID-19 pandemic, the wide use of biased risk prediction models is undoubtedly problematic.^28^ Access to care, including life-saving treatments, is the strongest predictor for, and a potential root cause of, poor health outcomes.^29^ Evidence also exists for substantial differences in health outcomes within an ethnic group depending on income and education.^30,31^ To add to the complexity surrounding this issue, there persists a debate whether race is a social or a biological concept;^32^ there are greater genetic differences between individuals of the same ethnic group than there are differences across ethnic groups. Furthermore, because socioeconomic factors might be distributed disproportionately, the inclusion of both ethnicity and socioeconomic parameters in health reporting has been recommended.^30,33^ A mere race adjustment might further the disparity in care.^34^

In addition to their use for triage purposes, these scoring systems are used for severity adjustment in research and for benchmarking performance. Our findings will also need to be taken into consideration for these purposes. For example, an ICU with a largely Black population would appear to be performing better than a unit of largely White patients on the basis of model mortality overpredictions for the Black people. For research, populations thought to be of equal severity might not be quite so. These are important considerations that will need to be addressed, but not of the urgency of the potential bias of systems used for triage purposes. Another important point is that given that MIMIC-III and eICU-CRD capture a wide variety of ICUs in the USA, these data should be potentially generalisable to most high-income settings where triaging of critical care resources on the basis of risk prediction tools have been discussed. However, a local assessment of model performances in different ethnic groups in different settings is needed.

There are a number of limitations of our study. First, patients were excluded from the analysis if they were missing data on ethnicity. Missing data is unfortunately an integral part of real-world clinical data analysis and, although extremely unlikely to be due to systematic bias, it is not possible to ascertain what resulted in the absence of the ethnic data in those patients. Second, the ascertainment of ethnicity was done at individual hospitals and was largely based on self-reporting. Third, the attribution of certain score components (eg, Glasgow coma scale) could be somewhat subjective. However, this issue is an inherent nature of ICU risk scoring and would be a factor in any study of similar nature. Fourth, the ethnic group category for Asian people is very heterogeneous, including Indian Asian people, Filipino Asian people, Chinese Asian people, and others. This categorisation might be imperfect, both biologically and socioeconomically, to group these ethnicities under the term Asian, and there might be significant differences to the performance of the scoring systems in these subgroups that would be lost after aggregation. Furthermore, there are relevant confounders that influence clinical outcome and there might be an unequal distribution of these variables across the groups. For example, Hispanic and Black populations were younger than the White and Asian populations in both the databases. However, some of the confounders are part of the models themselves (eg, APACHE IVa and OASIS) and therefore should be adjusted for in the output. In the current project, the purpose was to replicate what might happen at the bedside, where the clinicians do not adjust for any other confounders while applying a particular model in assessing risk. Lastly, the OASIS and SOFA analyses were not replicated on the newly released MIMIC-IV.

In conclusion, we found that the discrimination of the APACHE IVa, SOFA, and OASIS predictive models (ie, the ability of the model to differentiate between patients who survived and patients who died) differed between ethnicities at times, although no clear or systematic pattern emerged. However, when assessing calibration (ie, agreement between observed versus predicted risk), all of the prediction models systematically overestimated mortality across all ethnicities. Importantly, this poor level of calibration was most notable in Hispanic and Black patients and was found in all three scoring systems. In a world with health disparities and in which health-care providers’ triage decisions might be biased, current severity scoring prediction models might not be able to correctly and fairly characterise patient severity and risk. Incorporating precise socioeconomic and geographical parameters, along with a set of specific biomarkers for a given disease, into future prediction models might make such models less biased and more robust. Extreme care must be taken in the application of current scoring systems for triage decisions in individual patients, if they are to be used at all for these purposes in their present states.

## Supplementary Material

Supplementary Material

## Figures and Tables

**Table 1:** Patient characteristics by database

	All	Hispanic	Black	White	Asian	p value
**Number of patients**						
eICU-CRD	12 919	5057 (4·1%)	15 299 (12·4%)	100 694 (81·9%)	1869 (1·5%)	NA
MIMIC-III	43 823	1784 (4·1%)	4853 (11·1%)	35 997 (82·1%)	1189 (2·7%)	NA
**Age, years**						
eICU-CRD	64 (52–75)	62 (47–76)	58 (45–68)	65 (54–76)	65 (51–76)	<0·0001
MIMIC-III	64·5 (52–76)	53·3 (40·0–66·1)	58·1 (46·8–71·0)	63·8 (53·6–76·9)	61·3 (50·4–75·8)	<0·0001
**Sex, female**						
eICU-CRD	46%	46%	49%	45%	46%	<0·0001
MIMIC-III	43%	39%	56%	42%	42%	<0·0001
**APACHE IVa score**						
eICU-CRD	50 (37–67)	49 (36–67)	49 (35–67)	50 (37–67)	49 (36–68)	<0·0001
**OASIS score**						
MIMIC-III	30 (24–37)	29 (23–35)	30 (24–36)	30 (24–37)	30 (24–37)	<0·0001
**Expected mortality**						
eICU-CRD	11·5% (16)	12·2% (17)	11·9% (17)	11·4% (16)	11·8% (17)	0·0034
MIMIC-III	14·0% (14)	11·8% (12)	13·5% (14)	14·1% (14)	13·9% (14)	<0·0001
**Observed deaths**						
eICU-CRD	10 562 (8·6%)	442 (8·7%)	1219 (8·0%)	8732 (8·7%)	169 (9·0%)	0·0290
MIMIC-III	4847 (11·1%)	134 (7·5%)	443 (9·1%)	4114 (11·4%)	156 (13·1%)	<0·0001
**Length of stay in intensive care unit, days**						
eICU-CRD	1·8 (1·0–3·2)	1·67 (0·9–3·0)	1·9 (1·0–3·5)	1·8 (1·0–3·2)	1·8 (1·0–3·2)	<0·0001
MIMIC-III	2·1 (1·2–4·2)	2·0 (1·2–3·8)	2·1 (1·2–3·9)	2·1 (1·2–4·2)	2·1 (1·2–3·9)	<0·0001
**Duration on ventilator, days**						
eICU-CRD	2 (1–4)	2 (1–4)	2 (1–4)	2 (1–4)	2 (1–4)	<0·0001
MIMIC-III	2 (1–4)	2 (1–4)	2 (1–4)	2 (1–4)	2 (1–4)	<0·0001

Data are n, n (%), median (IQR), %, or mean (SD). eICU-CRD=electronic intensive care unit Collaborative Research Database. NA=not applicable. MIMIC-III=Medical Information Mart for Intensive Care III. APACHE IVa=Acute Physiology and Chronic Health Evaluation IVa. OASIS=Oxford Acute Severity of Illness Score.

**Table 2:** Deaths in different admission SOFA score ranges across ethnic groups in the eICU-CRD and MIMIC-III database

	All patients	Hispanic	Black	White	Asian	p value
**0–7**						
MIMIC-III (n=38 011)	2883 (7·6%)	74 (4·7%)	232 (5·6%)	2478 (7·9%)	99 (9·6%)	<0·0001
eICU-CRD (n=110 671)	6635 (6·0%)	262 (5·8%)	703 (5·2%)	5555 (6·1%)	115 (6·7%)	<0·0001
**8–11**						
MIMIC-III (n=4609)	1277 (27·7%)	30 (18·2%)	136 (24·4%)	1073 (28·5%)	37 (29·6%)	0·004
eICU-CRD (n=10 207)	2820 (27·6%)	126 (28·6%)	368 (26·4%)	2287 (27·8%)	39 (28·9%)	0·65
**>11**						
MIMIC-III (n=1203)	688 (57·2%)	30 (61·2%)	75 (56·8%)	563 (57·2%)	20 (54·1%)	0·92
eICU-CRD (n=2041)	1107 (54·2%)	54 (58·7%)	148 (49·5%)	890 (54·8%)	15 (57·7%)	0·20

Data are n (%). eICU-CRD=electronic intensive care unit Collaborative Research Database. MIMIC-III=Medical Information Mart for Intensive Care III. SOFA=Sequential Organ Failure Assessment.

## Research in context

### Evidence before this study

We searched PubMed on Sept 4, 2020, with no filter restrictions, using the terms, “intensive care unit severity scoring systems”, “bias”, and “racial bias” and found no results. These systems are used in critical care medicine for severity adjustment for research purposes and for benchmarking intensive care unit (ICU) performance. Ethnicity is generally documented in the process of hospital admission. However, none of the currently employed ICU severity scoring systems incorporate ethnicity or other relevant socioeconomic factors as a parameter in their analysis. We chose to examine three of these systems (Acute Physiology and Chronic Health Evaluation IVa, Oxford Acute Severity of Illness Score, and Sequential Organ Failure Assessment [SOFA]) for possible ethnically based bias. Out of the three scoring systems, SOFA has come to be used (in guidelines) for initial ICU triage purposes and to determine the continuation of mechanical ventilation in situations of limited resources during a pandemic.

### Added value of this study

We analysed the performance of three different clinical prediction models across four ethnicities in two large publicly available critical care databases. We found evidence that all three models overpredict mortality in all four groups. While this general phenomenon of model drift is already known, we show that the overprediction is more marked in Black people and Hispanic people, who have been historically more likely to have lower socioeconomic status compared with White people and Asian people in the USA. This was consistent in both the databases for all the prediction models tested.

In view of the aforementioned use of SOFA in the current pandemic for purposes of triage of potentially limited resources and the disparate clinical outcomes of particular ethnicities, we concluded that it is particularly important to ascertain whether severity scoring systems might contain previously undetected elements of bias, which would make them inappropriate to use for clinical decision making.

### Implications of all the available evidence

Triaging of critical care resources is being discussed widely in the context of the COVID-19 pandemic. To bring objectivity to the decision making process, clinical prediction scores have been proposed to form part of the triage process. SOFA is the most commonly proposed model in this context. We have shown evidence of bias in terms of the predicted versus observed mortalities (model calibration), such that their use should be approached with extreme caution, and it might be most prudent to avoid applying these prediction models to critical care triage across populations involving patients from different socioeconomic and ethnic backgrounds.
