# Supplementary material for: Performance of intensive care unit severity scoring systems across different ethnicities in the USA: a retrospective observational study
Source: Lancet Digit Health. Author manuscript; Available in PMC 2021 Apr 23. (PMC8063502; doi:10.1016/S2589-7500(21)00022-4)
Supplement: Supplementary Material [file NIHMS1689552-supplement-Supplementary_Material.pdf]

THE LANCET
Digital Health

Supplementary appendix

This appendix formed part of the original submission and has been peer reviewed. We post it as supplied by the authors.

Supplement to: Sarkar R, Martin C, Mattie H, Gichoya JW, Stone DJ, Celi LA. Performance of intensive care unit severity scoring systems across different ethnicities in the USA: a retrospective observational study. *Lancet Digit Health* 2021; **3:** e241–49.

Supplementary appendix

Github location of the codes: https://www.github.com/cjmartino/ITUscoringAnalysis

Summary of the three prediction scores in the study:

APACHE IVa
• Critical care mortality prediction model, predicts hospital mortality of the critically ill<br>• The model was developed using multivariate logistic regression.<br>• The model was built and tested in 104 ICUs in 45 U.S. based hospitals<br>• For a patient, a percentage probability of hospital mortality can be generated.<br>• Was developed based on 142 variables (including 116 admission categories and 17 acute physiological parameters), worst values in the first ICU day hours are taken for the acute physiology component<br>• Acute physiology contributes towards 65.9% of the score and age, chronic health condition, underlying diagnosis, ventilation status<br>• Currently often the clinical data is pulled from electronic health record automatically to generate the score. In some units, it can be done by the nurses.<br>• In can also predict ICU mortality
**OASIS**
• Critical care mortality prediction model, predicts hospital mortality and ICU mortality of the critically ill patients<br>• A machine learning technique called particle swarm optimization was used to develop the model.<br>• This was developed in 86 ICUs in 49 hospitals<br>• The model uses 10 patient parameters (heart rate, mean arterial pressure, temperature, respiratory rate, urine output, pre-ICU admission length of stay, GCS, age, being placed on a mechanical ventilator at any point during day 1 and admission following elective surgery.)<br>• Worst values on the first ICU days are taken<br>• A score is converted by logit to generate a probability of mortality
**SOFA**
• SOFA or Sequential Organ Failure and Assessment was built by expert consensus<br>• This was initially built for sepsis patients, but later on used for wider critical illnesses.<br>• SOFA can be calculated at admission and also thereafter daily with worst values for each

day.

- This is not build on a statistical model and cannot assign a specific mortality probability for a given score
- Six different organ system (cardiovascular, respiratory, hepatic, renal, coagulation and neurological) are evaluated
- Each system can be assigned a score between 1 and 4 depending on the relevant parameters
- It is widely used in clinical care and also in clinical trial settings

Additional discussion on risk categories of APACHE IVa and OASIS

Table S1 shows the trend of increasing SMR with increasing predicted mortality risk. This was the same in all ethnic groups, as shown in Figures S1 and S2. In the lower risk categories, SMR was markedly low in certain groups. For example, SMR was 0·47 in Hispanics in MIMIC-III within the 10-20% risk category. This pattern was the same in other risk strata in the eICU data as well (e.g. African Americans and Hispanics had SMRs of 0·49 and 0·31 respectively in the eICU data's 0-5% risk category). An exception to this trend of improving SMR with increasing predicted risk was African Americans in MIMIC-III, where no such improvement was seen and the patients within this group had persistently low SMRs (<0·7) in all risk categories.

Supplementary table S1: SMR across risk categories in different ethnic groups

**Risk categories**	Dataset	Hispanic	African American	White	Asian
**0-5%**	eICU	0·31	0·49	0·51	0·54
	MIMIC	0·55	0·72	0·95	0·65
**5-10%**	eICU	0·66	0·65	0·69	0·76
	MIMIC	0·63	0·72	0·75	0·67
**10-20%**	eICU	0·72	0·61	0·77	0·89
	MIMIC	0·47	0·54	0·74	0·92
**20-50%**	eICU	0·71	0·67	0·79	0·65
	MIMIC	0·64	0·67	0·8	0·85
**50-100%**	eICU	0·90	0·77	0·85	0·90
	MIMIC	0·92	0·68	0·86	0·96

Supplementary figure 1: SMR trend in different risk categories in all ethnic groups in eICU database

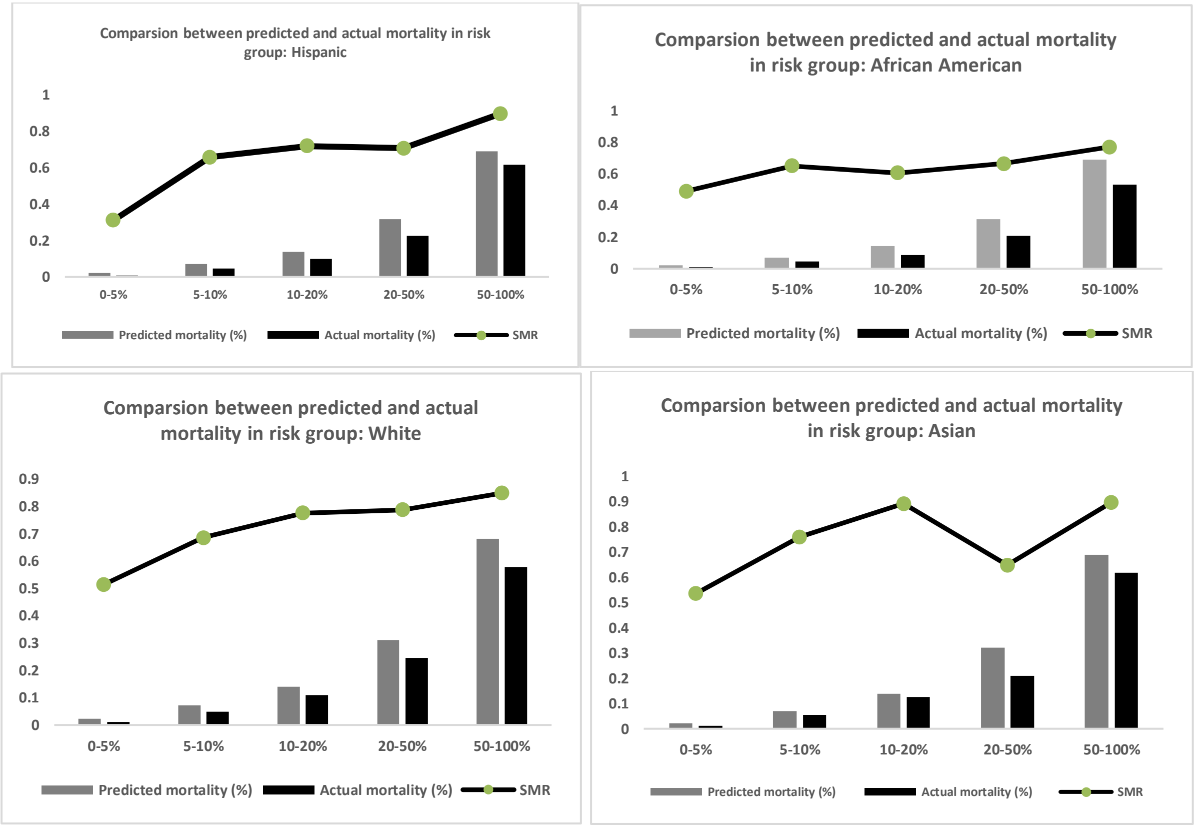

Supplementary figure 2: Trend in different risk categories in all ethnic groups in MIMIC-III database

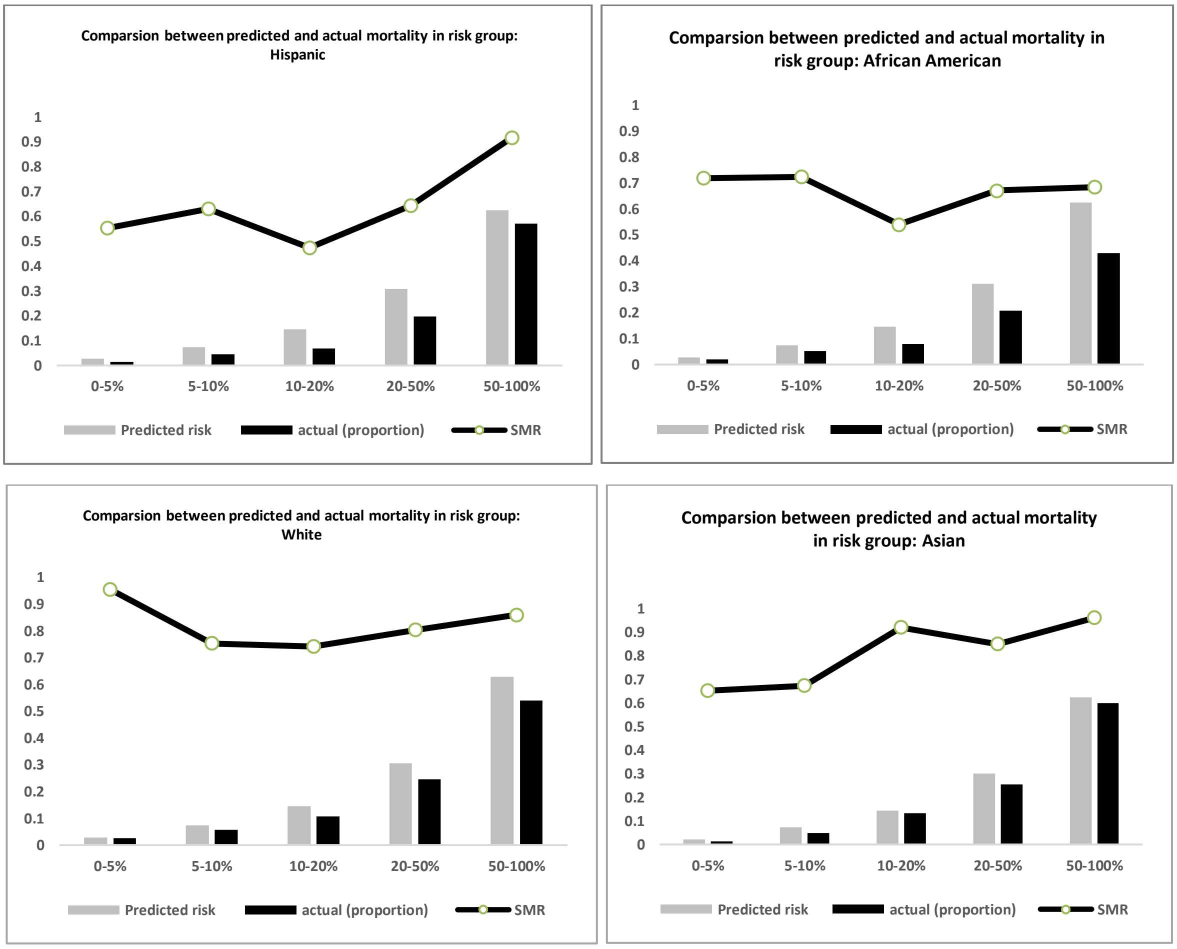

Supplementary table S2: AUROC in different ethnicities in both the databases (with 95% CIs)

Scoring system (Database)	Hispanic	African American	White	Asian	p-value
APACHE IVa (eICU)	0·89 (0·8744, 0·903)	0·87 (0·857, 0·878)	0·86 (0·860, 0·868)	0·86 (0·831, 0·889)	0·02
OASIS (MIMIC-III)	0·76 (0·72,0·81)	0·75 (0·73,0·78)	0·76 (0·75,0·77)	0·77 (0·73, 0·81)	0·85

Supplementary table S3: Predicted and observed mortality in different ethnic groups along (SMR = actual/predicted mortality ratio in each patient group); SMRs in eICU are for APACHE IVa and SMRs for MIMIC-III are for OASIS scores respectively.

Ethnicity	Dataset	Total number	Mean predicted mortality	Predicted deaths	Actual deaths	SMR
Hispanic	eICU	5057	0·12 (0.17)	608	442	0·73
	MIMIC-III	1784	0·12 (0.12)	210	134	0·64
African American	eICU	15299	0·12 (0.17)	1813	1219	0·67
	MIMIC-III	4853	0·14 (0.14)	657	443	0·68
White	eICU	100694	0·11(0.16)	11456	8732	0·76
	MIMIC-III	35997	0·14 (0.14)	5081	4114	0·81
Asian	eICU	1869	0·12 (0.17)	220	169	0·77
	MIMIC-III	1189	0·14 (0.14)	165	156	0·95
p-value (across group difference)	eICU					<0·0001
	MIMIC					<0·0001

Supplementary table S4: Ratio of observed mortality in each group to overall mortality by admission SOFA category and ethnic group in both databases.

SOFA score	Database	Hispanic	African American	White	Asian
0-7	MIMIC	0·62	0·74	1·04	1·6
	eICU	0·96	0·86	1·02	1·12
8-11	MIMIC	0·66	0·88	1·03	1·07
	eICU	1·04	0·95	1.00	1·05
>11	MIMIC	1·07	0·99	1	0·95
	eICU	1·08	0·91	1·01	1·06

Supplementary figure 3:

Top panel: Forest plot for AUROCs from the eICU-CRD. There is a clear separation between the White and Hispanic groups. All other group pairs have overlapping 95% confidence intervals (CIs). Bottom panel: Forest plot for AUROCs from the MIMIC-III. The absolute AUROCs are similar, with overlapping 95% CIs for all groups.

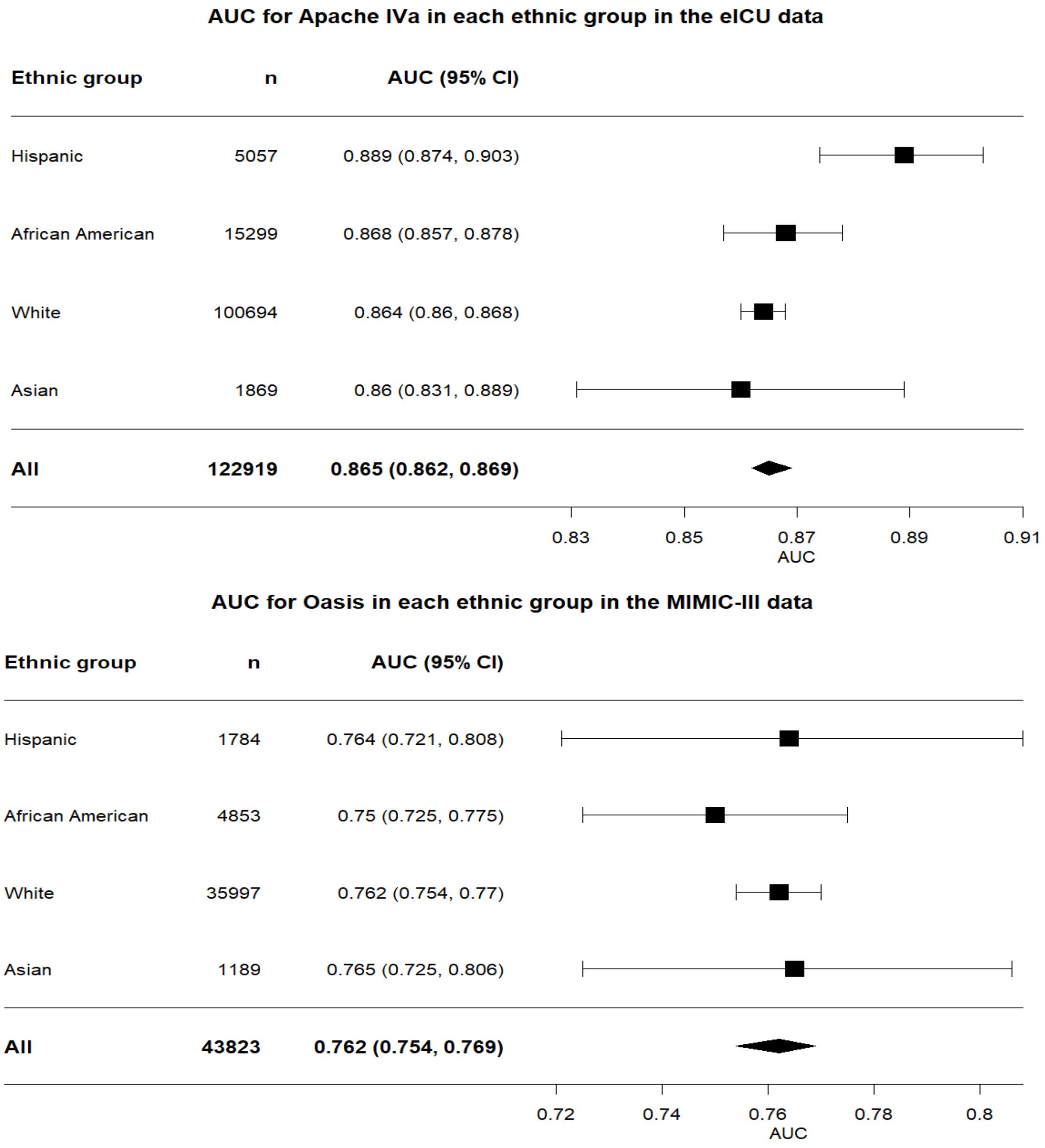

Supplementary figure 4:

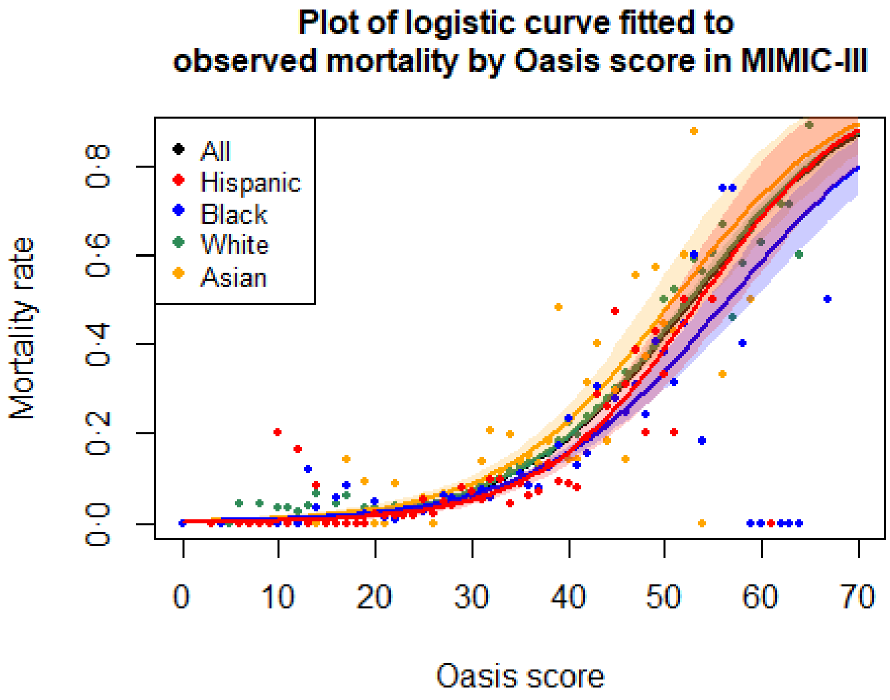

Supplementary figure 5:

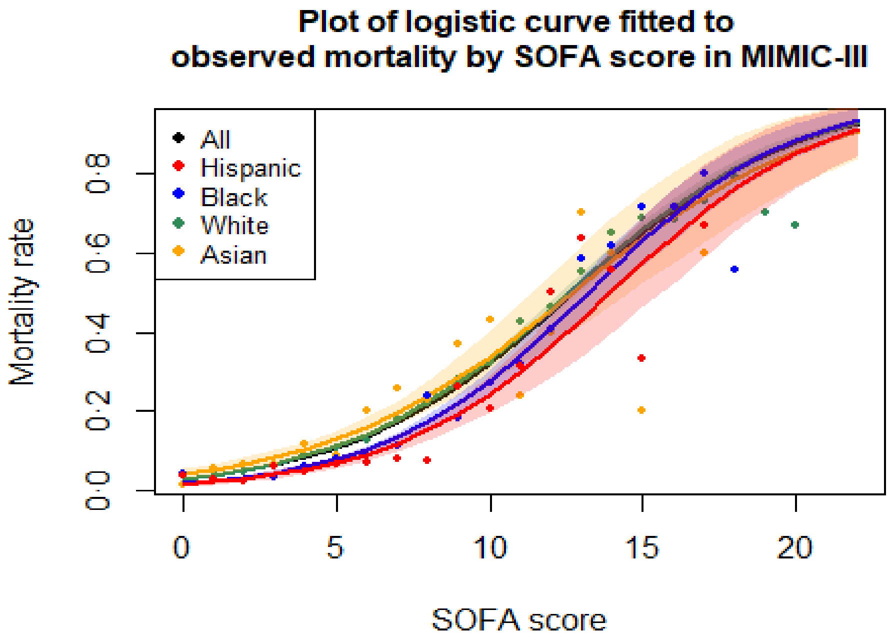

Supplementary figure 6:

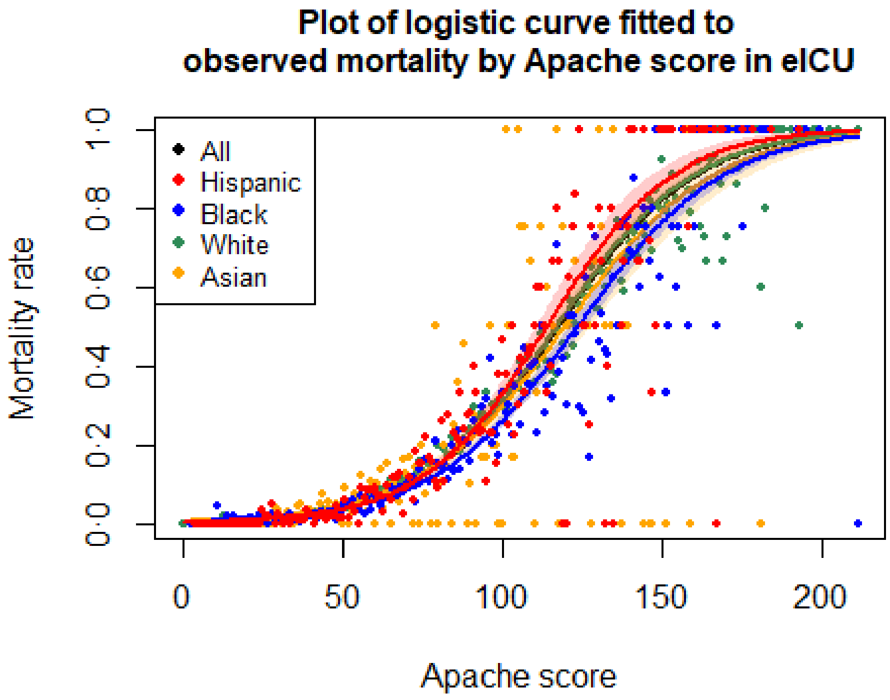

Supplementary figure 7:

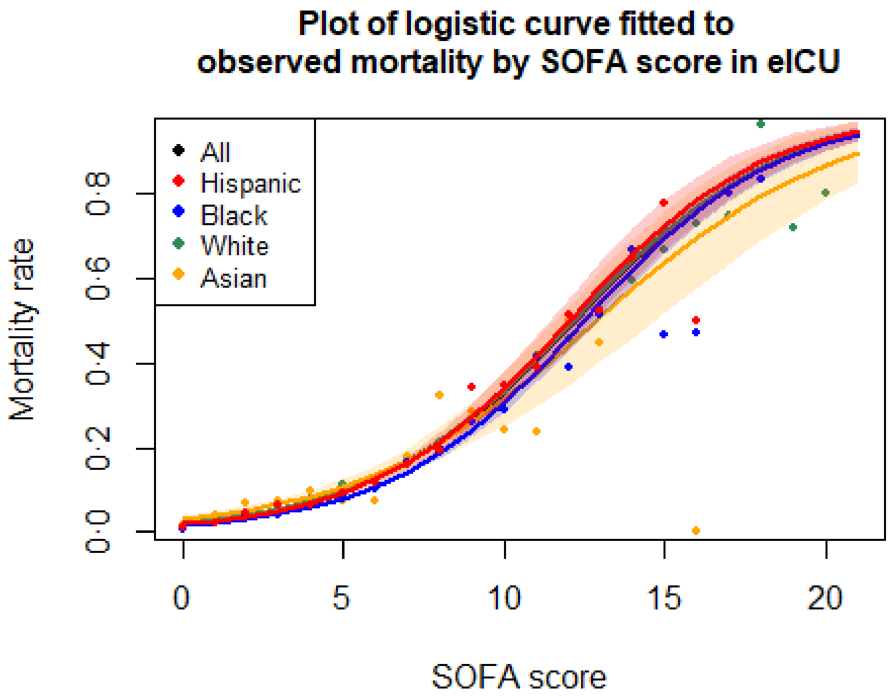

Supplementary figure 8:

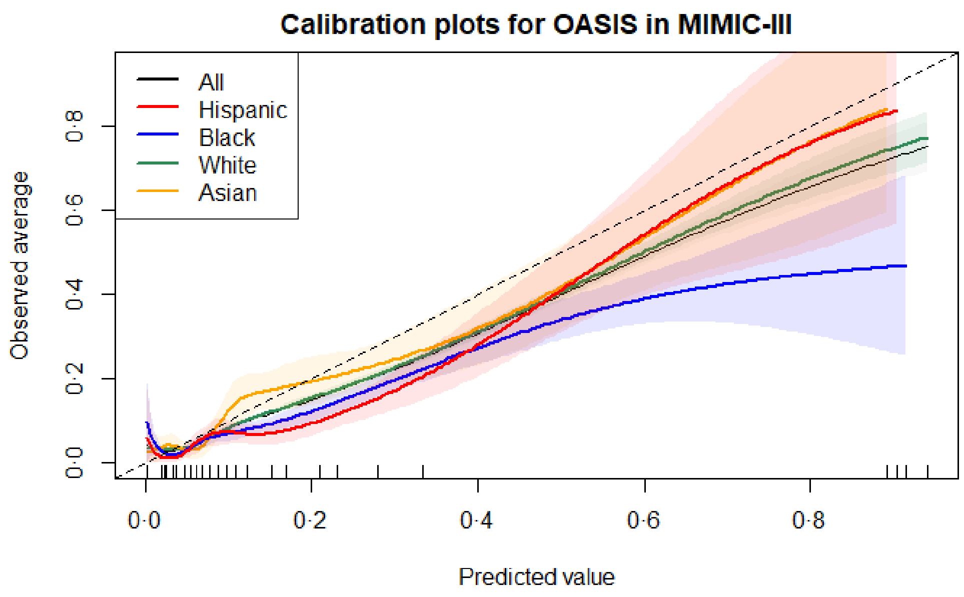

Supplementary figure 9:

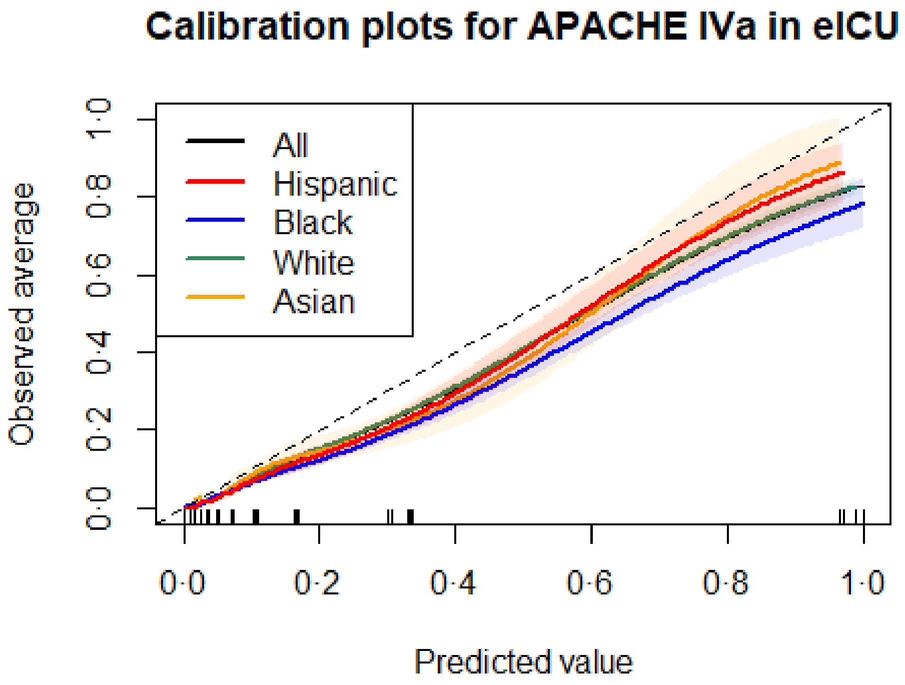

Explanatory power of ethnicity and predictive scoring

Supplementary table S5: Logistic regression of APACHE IVa score and in-hospital death (eICU)

	Estimate	Std. Error	z value	Pr(>|z|)
(Intercept)	-5·519	0·034	-164·583	0
apachescore	0·047	0·000	114·491	0

R^2 = 0·1972056.

Supplementary table S6: Logistic regression of OASIS score and in-hospital death(MIMIC-III)

	Estimate	Std. Error	z value	p-value
(Intercept)	-5·890	0·071	-83·531	<0·0001
OASIS	0·111	0·002	60·054	<0·0001

R^2 = 0.1236887

Supplementary table S7: Logistic regression of admission SOFA score and in-hospital death (MIMIC-III)

	Estimate	Std. Error	z value	p-value
(Intercept)	-3·472	0·032	-108·508	<0·0001
SOFA	0·273	0·005	58·945	<0·0001

R^2 = 0·11774

Supplementary table S8: Logistic regression of SOFA score and in-hospital death (eICU)

	Estimate	Std. Error	z value	Pr(>|z|)
(Intercept)	-3·821	0·021	-179·985	0
SOFA1	0·313	0·003	97·837	0

R^2 = 0·1177927.

Supplementary table S9: Logistic regression of ethnicity and in-hospital death (eICU), with African American as baseline ethnicity

	Estimate	Std. Error	z value	Pr(>|z|)
(Intercept)	-2·447	0·030	-81·951	0·000
Asian	0·138	0·086	1·607	0·108
White	0·092	0·032	2·896	0·004
Hispanic	0·101	0·058	1·739	0·082

R^2 = 7·318419710^{-5}.

Supplementary table S10: Logistic regression of ethnicity and in-hospital death (MIMIC-III), with Asian as baseline ethnicity

	Estimate	Std. Error	z value	p-value
(Intercept)	-1·890	0·086	-22·007	<0·0001
African American	-0·408	0·099	-4·105	<0·0001
Hispanic	-0·620	0·124	-4·991	<0·0001
White	-0·157	0·087	-1·798	0·072

R^2 = 0·0011754

Supplementary table S11: Logistic regression of ethnicity and in-hospital death controlled for APACHE IVa score (eICU), with African American as baseline ethnicity

	Estimate	Std. Error	z value	Pr(>|z|)
(Intercept)	-5·689	0·048	-119·610	0·000
Asian	0·208	0·098	2·125	0·034
White	0·186	0·036	5·109	0·000
Hispanic	0·184	0·066	2·778	0·005
Apache score	0·047	0·000	114·483	0·000

R^2 = 0·1976472.

Supplementary table S12: Logistic regression of ethnicity and in-hospital death controlled for OASIS score (MIMIC-II), with Asian as baseline ethnicity

	Estimate	Std. Error	z value	p-value
(Intercept)	-5·654	0·114	-49·391	<0·0001
African American	-0·434	0·106	-4·085	<0·0001
Hispanic	-0·500	0·132	-3·802	<0·0001
White	-0·202	0·094	-2·156	0·031
OASIS	0·111	0·002	59·904	<0·0001

R^2 = 0.1246951

Supplementary table S13: Logistic regression of ethnicity and in-hospital death controlled for SOFA score (eICU), with African American as baseline ethnicity

	Estimate	Std. Error	z value	Pr(>|z|)
(Intercept)	-4·005	0·037	-107·052	0·000
ethnicityAsian	0·317	0·092	3·460	0·001
ethnicityCaucasian	0·205	0·034	6·013	0·000
ethnicityHispanic	0·168	0·062	2·711	0·007
SOFA1	0·314	0·003	97·937	0·000

R^2 = 0·1177927.

Supplementary table S14: Logistic regression of ethnicity and in-hospital death controlled for admission SOFA score (MIMIC-III), with Asian as baseline ethnicity

	Estimate	Std. Error	z value	p-value
(Intercept)	-3·282	0·096	-34·102	<0·0001
African American	-0·499	0·106	-4·693	<0·0001
Hispanic	-0·638	0·133	-4·801	<0·0001
White	-0·147	0·094	-1·568	0·117
SOFA	0·274	0·005	59·050	<0·0001

R^2 = 0·119408

The contribution of the risk scores and ethnicity to variation in in-hospital mortality in the eICU-CRD and MIMIC-III databases.

The Oasis score explains 12·367% of the variation in mortality, and the ethnicity explains 0·115% in MIMIC-III.

The APACHE IVa score explains 19·759% of the variation in mortality, and the ethnicity explains 0·055% in eICU-CRD

The SOFA score explains 11·837% of the variation in mortality, and the ethnicity explains 0·189% in the MIMIC-III database.

The SOFA score explains 11·801% of the variation in mortality, and the ethnicity explains 0·032% in the eICU-CRD database.
